# Intravenous Polymyxin B as Adjunctive Therapy to High-Dose Tigecycline for the Treatment of Nosocomial Pneumonia Due to Carbapenem-Resistant *Acinetobacter baumannii* and *Klebsiella pneumoniae*: A Propensity Score-Matched Cohort Study

**DOI:** 10.3390/antibiotics12020273

**Published:** 2023-01-30

**Authors:** Lei Zha, Xue Zhang, Yusheng Cheng, Qiancheng Xu, Lingxi Liu, Simin Chen, Zhiwei Lu, Jun Guo, Boris Tefsen

**Affiliations:** 1 Department of Respiratory Medicine, The First Affiliated Hospital of Wannan Medical College (Yijishan Hospital of Wannan Medical College), Wuhu 241000, China; 2 Institute of Infection and Global Health, University of Liverpool, Liverpool L69 7BE, UK; 3 Department of Intensive Care Unit, West China Hospital, Sichuan University, Chengdu 610041, China; 4 Department of Critical Care Medicine, The First Affiliated Hospital of Wannan Medical College (Yijishan Hospital of Wannan Medical College), Wuhu 241000, China; 5 Division of Microbiology, Department of Biology, Utrecht University, 3584 CH Utrecht, The Netherlands; 6 Natural Sciences, Ronin Institute, Montclair, NJ 07043, USA

**Keywords:** *Acinetobacter baumannii*, carbapenem resistance, *Klebsiella pneumoniae*, nosocomial infection, pneumonia, polymyxin, tigecycline

## Abstract

Although the combination of polymyxin and tigecycline is widely used in treating carbapenem-resistant bacterial infections, the benefit of this combination is still uncertain. To assess whether adding polymyxin B to the high-dose tigecycline regimen would result in better clinical outcomes than the high-dose tigecycline therapy in patients with pneumonia caused by carbapenem-resistant *Klebsiella pneumoniae* and *Acinetobacter baumannii*, we conducted a propensity score-matched cohort study in a single center between July 2019 and December 2021. Of the 162 eligible patients, 102 were included in the 1:1 matched cohort. The overall 14-day mortality in the matched cohort was 24.5%. Compared with high-dose tigecycline, the combination therapy was not associated with better clinical outcomes, and showed similar 14-day mortality (OR, 0.72, 95% CI 0.27–1.83, *p* = 0.486), clinical cure (OR, 1.09, 95% CI 0.48–2.54, *p* = 0.823), microbiological cure (OR, 0.96, 95% CI 0.39–2.53, *p* = 0.928) and rate of nephrotoxicity (OR 0.85, 95% CI 0.36–1.99, *p* = 0.712). Subgroup analyses also did not demonstrate any statistical differences. Based on these results, it is reasonable to recommend against adding polymyxin B to the high-dose tigecycline regimen in treating pneumonia caused by carbapenem-resistant *K. pneumoniae* and *A. baumannii*.

## 1. Introduction

Carbapenem-resistant Gram-negative bacteria, including *Acinetobacter baumannii*, *Pseudomonas aeruginosa*, and Enterobacteriaceae such as *Klebsiella pneumoniae*, *Escherichia coli*, and *Enterobacter* spp. have been prioritized by the World Health Organization (WHO) as a critical group of pathogens that requires new antibiotics due to their increasing prevalence and extremely limited therapeutic options [1]. Data from The European Antimicrobial Resistance Surveillance Network 2017 (EARS-Net 2017) indicated that among the top ten pathogens causing intensive care unit (ICU) acquired pneumonia, the carbapenem-resistance rate in *Acinetobacter* spp. and *Klebsiella* spp. was as high as 64% and 15%, respectively [2]. The disease burden of infections caused by these resistant pathogens has also increased significantly. According to the results from a population-level modelling analysis using data from the EARS-Net, the proportion of the disability-adjusted life-years (DALYs) due to carbapenem-resistant bacteria increased from 18% in 2007 to 28% in 2015, while the DALYs due to carbapenem-resistant *K. pneumoniae* doubled during the same period (from 4.3% to 8.79%) [3].

Mortality in patients with pneumonia, one of the most common diseases resulting from infection by carbapenem-resistant pathogens, is worryingly high. A recent study including 690 critically ill patients with nosocomial pneumonia caused by carbapenem-resistant Gram-negative bacteria reported in-hospital mortality of 46.1% [4]. Although the mortality was slightly lower in a multicenter study conducted in 18 hospitals in the US, carbapenem resistance still contributed to 27% of excess hospital mortality in patients with pneumonia [5]. The excess mortality could be partially attributed to inappropriate antibiotic therapy. A study indicated that patients with nosocomial pneumonia caused by carbapenem-resistant Gram-negative bacteria were more likely to receive inappropriate antibiotic therapy than their susceptible counterparts (25.78% vs. 10%) [6]. Moreover, with limited antibiotic choices, targeted therapies for such infections are usually restricted to suboptimal agents, potentially leading to worse clinical outcomes as well. 

Although there are new antibiotics marketed in recent years, the availability of these drugs in certain regions is still insufficient. According to data reported in a nationwide survey in China in 2020, among the 212 participating hospitals, only 16% of hospitals routinely offered the only marketed new β-lactam/β-lactamase inhibitor ceftazidime-avibactam [7]. Consequently, old antibiotics, like tigecycline and polymyxins, turn to be the mainstream therapy for infections caused by carbapenem-resistant Gram-negative bacteria [8,9]. Although tigecycline and polymyxins present good in vitro activity against carbapenem-resistant bacteria, the clinical efficacy of these old antibiotics is still unsatisfactory [10,11]. Compared with ceftazidime-avibactam, colistin therapy resulted in higher mortality and lower clinical response in patients with carbapenem-resistant Enterobacteriaceae infections [12]. Similarly, studies also demonstrated that tigecycline-containing treatment was associated with increased mortality [13,14]. Under such circumstances, using two in vitro active antibiotics together is widely accepted, especially in patients with severe infections.

Due to the good in vitro synergistic effect of tigecycline and polymyxins [15,16], combination therapy with these two drugs has become one of the most preferred regimens in treating infections caused by carbapenem-resistant *A. baumannii* and *K. pneumoniae* [17]. In China, even with the limited availability of polymyxins in some hospitals, tigecycline plus polymyxin was still the third most frequent therapeutic choice for carbapenem-resistant infections, after tigecycline plus cefoperazone-sulbactam and tigecycline plus carbapenem [7]. Despite the wide use of this combination, clinical studies demonstrating its efficacy are still limited. As of writing, only three studies assessed its effectiveness in treating infections (bacteremia and intra-abdominal infection) due to carbapenem-resistant *A. baumannii*, and none of them demonstrated any benefit [18,19,20]. Moreover, in patients with the minimum inhibitory concentration (MIC) of tigecycline against *A. baumannii* greater than 2 mg/L, the combination of tigecycline and colistin was even associated with an increased 14-day mortality in comparison with colistin-carbapenem therapy (hazard ratio 6.93, 95% CI, 1.61–29.78, *p* = 0.009) [19].

It should be noted that the tigecycline used in each of these studies was the standard dose (100 mg loading dose following 50 mg per 12 h) rather than the recommended high dose (200 mg loading dose following 100 mg per 12 h). Pharmacokinetic studies have demonstrated that using the standard dose of tigecycline resulted in suboptimal concentrations in blood (0.72 mg/L) and lung (0.34 mg/L), which is insufficient in controlling infections caused by those carbapenem-resistant pathogens, given MICs of the contemporary clinical isolates [21,22,23]. Moreover, the synergistic effect of colistin and tigecycline has shown to be dose dependent. When colistin was combined with a high concentration of tigecycline, the bactericidal effect increased, and the bactericidal effect was attenuated when combined with the low concentration of tigecycline [24]. Taken together, the use of the standard dose of tigecycline in the combination regimen might be a probable reason for the lack of differences in outcomes between colistin and colistin-tigecycline combination therapy in previous studies. 

However, it is still unclear whether using the high-dose regimen of tigecycline could offset the disadvantages mentioned above. Compared with the standard dose of tigecycline, the high-dose regimen increased the probability of target attainment at MICs of 1 and 2 mg/L from 72% to 99% and 11% to 71%, respectively [25]. Moreover, favorable clinical outcomes of the high-dose tigecycline in treating severe infections have been demonstrated in observational studies [26,27]. Therefore, we hypothesized that combining high-dose tigecycline with polymyxin might improve clinical outcomes. In the present study, we assessed whether adding polymyxin B to the high-dose tigecycline would result in better clinical outcomes than that of the high-dose tigecycline therapy in patients with pneumonia due to carbapenem-resistant *K. pneumoniae* and *A. baumannii*.

## 2. Methods

### 2.1. Study Design

A retrospective cohort study was conducted in the West China Hospital, Sichuan University. Patients admitted to the intensive care unit between July 2019 and December 2021 with the diagnosis of nosocomial pneumonia were reviewed. The institutional review board of the West China Hospital approved the study, and the patient’s consent was obtained from a family member or authorized person (reference number 2019-843).

### 2.2. Definition and Diagnosis of Pneumonia

The diagnosis of nosocomial pneumonia, including hospital-acquired pneumonia (HAP) and ventilator-associated pneumonia (VAP), was made based on the 2016 clinical practice guidelines by the Infectious Diseases Society of America and the American Thoracic Society [28]. In brief, patients with a new or progressive infiltrate, consolidation, cavitation, or pleural effusion on their chest radiographs along with two or more of the following criteria were considered pneumonia: fever (>38 °C) or hypothermia (<35.5 °C), leukocytosis (>10 × 10^12^/L) or leukopenia (<4 × 10^12^/L), newly onset or worsening cough with purulent sputum or aspirate, and deteriorated oxygenation that required an increment in oxygen or ventilation support. 

Ventilator-associated pneumonia was defined as pneumonia developed more than 48 h after intubation. Hospital-acquired pneumonia was pneumonia not incubating at the time of hospital admission but occurred 48 h or more after the admission. Pathogens responsible for the corresponding nosocomial pneumonia were determined by the quantitative or semi-quantitative culture of specimens from bronchoalveolar lavage, endotracheal aspirate, or sputum, collected within 48 h before or after the onset of pneumonia [29]. All endotracheal aspirate and sputum samples were subjected to microscopic analysis; only specimens with more than 25 neutrophils and less than ten squamous epithelial cells per low-power field were considered qualified specimens for culture [30]. The threshold of quantitative culture for a positive bronchoalveolar lavage and endotracheal aspirate is 10^4^ CFU/mL and 10^5^ CFU/mL, respectively; the threshold for the semi-quantitative culture of sputum is at least moderate growth on plates [31].

### 2.3. Microbiological Tests

The identification of pathogens was performed with the Vitek 2 system (bioMérieux) and matrix-assisted laser desorption/ionization time-of-flight mass spectrometry (bioMérieux). Antimicrobial susceptibility testing was performed with the microdilution method and interpreted according to the breakpoint recommended by The European Committee on Antimicrobial Susceptibility Testing (EUCAST) [32]. Pathogens with the MIC of meropenem ≥ 8 mg/L were defined as carbapenem-resistant, and those with the MIC of tigecycline and polymyxin B ≤ 2 mg/L were considered susceptible to tigecycline and polymyxins, respectively. 

### 2.4. Participants and Antimicrobial Therapy

Patients aged over 18 years with the diagnosis of nosocomial pneumonia caused by carbapenem-resistant *K. pneumoniae* or *A. baumannii* receiving either tigecycline or tigecycline-polymyxin B as their targeted therapy within 3 days after the report of the responsible pathogen were eligible for inclusion in the present study. Tigecycline used in the present study was at its high-dose regimen (200 mg loading dose following 100 mg per 12 h). Polymyxin B was administrated with a loading dose of 1,000,000 IU following 750,000 IU per 12 h. In patients with renal impairment, the maintenance dose of polymyxin B was adjusted to 500,000 IU per 12 h. Combination therapy with carbapenems, aminoglycosides, fluoroquinolones, and classical β-lactam/β-lactamase inhibitors (piperacillin-tazobactam, cefoperazone-sulbactam) was allowed, while the combination with ceftazidime-avibactam or minocycline was excluded in the present study. Only patients who received polymyxin B for more than 50% of the total tigecycline treatment time were treated as the combination therapy. Polymicrobial infections were permitted if the concomitant pathogens were susceptible to tigecycline. Patients meeting the following criteria were excluded: patients received the targeted therapy less than 48 h, including patients who died and those who received the change of antibiotics within 48 h; pathogens had the MIC of tigecycline or polymyxin B > 2 mg/L; concomitantly had other site infections during the pneumonia course, like intra-abdominal infection, central nervous system infection, central catheter infections, urinary tract infection and wound infections; co-infected with *P. aeruginosa* or *Stenotrophomonas maltophilia*; co-infected with Gram-positive bacteria or fungi. In the case of patients with multiple nosocomial pneumonia, only the first admission was included.

### 2.5. Clinical Outcomes and Definitions

The primary interest of the study was the 14-day all-cause mortality after the onset of pneumonia. The secondary outcomes were the 14-day clinical cure, microbiological cure, and nephrotoxicity occurring during the targeted antibiotic course. Clinical cure was defined as the complete resolution of symptoms and signs due to pneumonia or such improvement of patients that antibiotics were stopped within 14 days after the onset of pneumonia. The microbiological cure was defined as the absence of responsible pathogens recovered from cultures of the sputum, endotracheal aspirate or the bronchoalveolar lavage within 14 days after the initiation of the targeted therapy. Nephrotoxicity was defined as an increment in serum creatinine of 0.5 mg/dL from the baseline to at least two consecutive measurements during the antibiotic course after receiving 2 or more days of the targeted therapy [19]. For patients who died within 14 days, the follow-up time point of the assessment was set to the date of death.

### 2.6. Data Extraction 

The following information was collected from the patient’s medical record: age, gender, preexisting medical conditions, type of pneumonia, responsible pathogens, antimicrobial regimens, duration of the targeted antibiotic therapy, whether having septic shock, whether receiving vasopressors, the sequential organ failure assessment (SOFA) score, the 14-day mortality, clinical and microbiology cure within 14 days after the onset of pneumonia, and nephrotoxicity. Moreover, the age-adjusted Charlson comorbidity index score was calculated based on information from the medical records.

### 2.7. Statistical Analysis

Categorical variables were summarized as counts and percentages. The differences in categorical variables between patients in the tigecycline group and the tigecycline-polymyxin B group were analyzed with the Chi-square test or Fisher’s exact test. Continuous variables were expressed as median and interquartile ranges and compared with the Mann–Whitney U test. 

The propensity score matching was applied to identify a cohort with similar baseline characteristics in the two groups. The propensity score was calculated using the multivariable logistic regression model, with receiving the combination therapy as the dependent variable and a priori decided variables (the age-adjusted Charlson comorbidity index score, inappropriate initial antibiotic therapy, pathogen, polymicrobial pneumonia and the SOFA score) as covariates. The matched cohort was created with the 1:1 matching protocol through a greedy-matching algorithm, with a caliper of 0.2 of the standard deviation of the logit of the propensity score. Standardized mean differences of baseline variables were calculated to assess the balance in the matched cohort. Odds ratios (OR) and 95% confidence intervals (CI) for clinical outcomes were calculated by adjusting the SOFA score, polymicrobial infection, inappropriate initial antibiotic therapy, and the age-adjusted Charlson comorbidity index score in the matched cohort. The same analyses were also conducted in the original cohort as the sensitivity analysis. The discrimination of the multivariable logistic regression model of the primary outcome was assessed with the area under the receiver operating characteristic curve (AUC).

Subgroup analyses of the primary outcome were also conducted in the matched cohort. Patients were stratified with age (≤65 or >65 years), pneumonia type (HAP or VAP), pathogen (*A. baumannii* or *K. pneumoniae*), and initial empirical antibiotic therapy (appropriate or inappropriate). Moreover, we also conducted sensitivity analyses in patients without polymicrobial pneumonia and septic shock. The OR and 95% CI of the primary outcome in each subgroup were calculated in the univariable logistic regression model by defining patients receiving tigecycline as the reference. All reported *p* values were two-sided, and the *p* < 0.05 was considered statistically significant. All statistical analyses were performed with R software version 3.6.2 (R Foundation for Statistical Computing).

## 3. Results

### 3.1. Study Cohort

**Figure 1:**
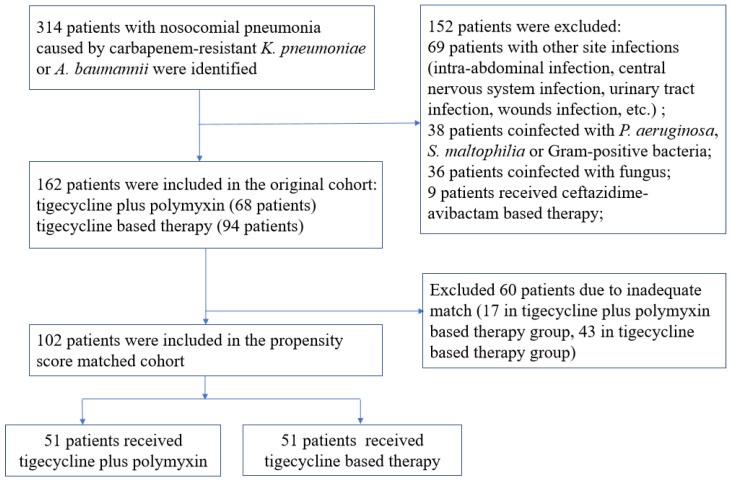
Flowchart of the study inclusion process.

There were 314 patients with nosocomial pneumonia due to carbapenem-resistant *A. baumannii* and *K. pneumoniae* identified from medical records, among whom 152 met the exclusion criteria. Of the remaining 162 patients in the original study cohort, 68 (42%) received the combination therapy, and 94 (58%) received tigecycline therapy (Figure 1). 

**Figure 2:**
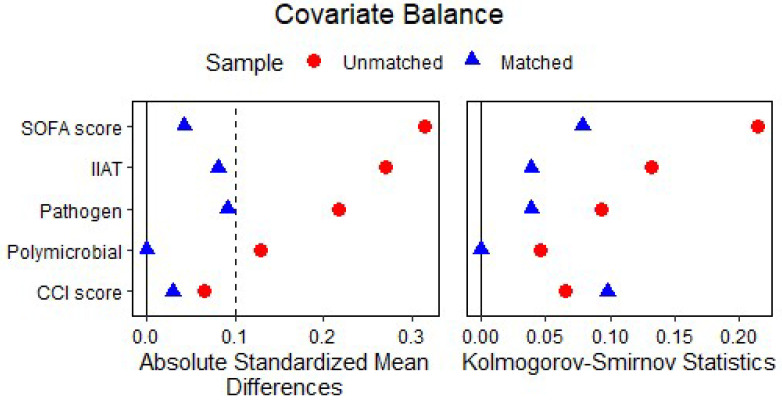
The covariate balance in the two groups before and after the propensity score matching. Red dots and blue triangles indicated the absolute standardized mean differences (**left**) and the Kolmogorov–Smirnov statistics (**right**) of the covariate in the original and the matched cohort, respectively. SOFA, Sequential Organ Failure Assessment; IIAT, Inappropriate Initial Antibiotic Therapy; CCI score, the age-adjusted Charlson comorbidity index.

There were imbalances in the prespecified baseline variables between the two groups in the original cohort, such as the inappropriate initial antibiotic therapy, responsible pathogen, polymicrobial pneumonia and the SOFA score, which have been demonstrated well to be associated with clinical outcomes in patients with severe pneumonia. After propensity score matching, 51 patients receiving the combination therapy were matched with 51 patients receiving tigecycline (Figure 1). The absolute standardized mean differences of the prespecified variables were then less than 0.1 in the matched cohort, indicating acceptable minor differences between the two groups (Figure 2).

### 3.2. Characteristics of Patients in the Matched Cohort

**Figure 3:**
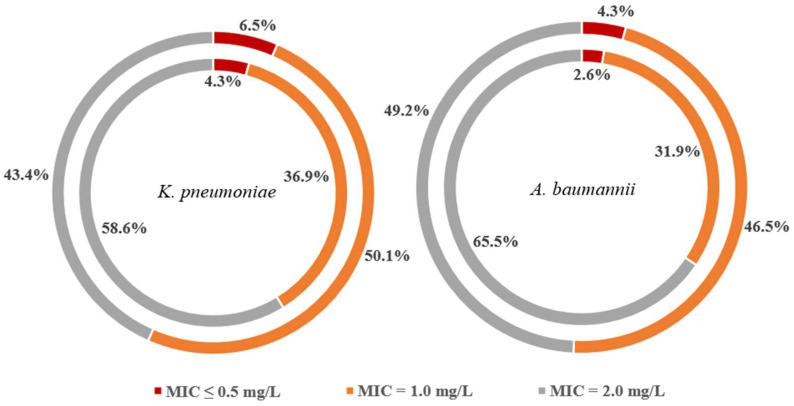
The distribution of minimum inhibitory concentration values of tigecycline and polymyxin B in *A. baumannii* and *K. pneumoniae* strains included in the study. The outer cycle represents the distribution of polymyxin B MICs, while the inner cycle is the distribution of tigecycline MICs. MIC, minimum inhibitory concentration.

The median age of patients in the matched cohort was 60 (IQR 51.25–76) years, and 69.6% were male. Most patients reported comorbidities, with an age-adjusted Charlson comorbidity index score of four (IQR 2–6). In the matched cohort, apart from 19 patients having malignancy, no other high-risk patients were included (e.g., patients suffering from cystic fibrosis, infected with human immunodeficiency virus, or having received an organ transplantation). A total of 63 (61.8%) patients were diagnosed with VAP, and 49 (38.2%) were diagnosed with HAP. Among the included patients, 70.6% (72) were infected with *A. baumannii*, 29.6% (30) were with *K. pneumoniae*, and 9.8% (10) of them were polymicrobial pneumonia. The distribution of MICs of tigecycline and polymyxin B in *A. baumannii* and *K. pneumoniae* strains is shown in Figure 3. At the onset of pneumonia, patients were critically ill, with a SOFA score of nine (IQR 7.25–12). 87.3% (89) of patients developed septic shock during the pneumonia course, and the median time of using vasopressors was 10 (IQR 6–18) days. 

In terms of antibiotic therapy, less than half of patients (47%, 48) received appropriate initial antibiotic treatment. For the targeted therapy, 83.3% (85) of patients received concomitant antibiotics in addition to tigecycline or tigecycline-polymyxin B, of which carbapenems and cefoperazone-sulbactam were the most frequently used concomitant antibiotics. The duration of targeted antibiotic therapy was relatively long in the matched cohort, with a median time of 15 (IQR 10–25) days. Details of patients’ characteristics in the original and the matched cohort are summarized in Table 1. 

### 3.3. Clinical Outcomes

The overall 14-day mortality, clinical cure, and microbiological cure in the matched cohort were 24.5% (25/102), 53.9% (55/102), and 27.7% (28/102), respectively. Nephrotoxicity developed in 50 (49%) patients during the targeted antibiotic course. In the multivariable logistic regression analysis, patients receiving the combination therapy were not associated with better clinical outcomes than those receiving tigecycline (AUC of the model of the primary clinical outcome was 0.71, 95% CI 0.58–0.83). Moreover, the adjunctive therapy of polymyxin B to high-dose tigecycline did not increase the rate of nephrotoxicity (adjusted OR 0.85, 95% CI 0.36–1.99, *p* = 0.712). Similar results were also shown in the original study cohort that was run as the sensitivity analyses (AUC of the model of the primary clinical outcome was 0.73, 95% CI 0.63–0.83) (Table 2).

### 3.4. Subgroup Analyses

Among the predefined subgroup analyses, patients receiving the combination therapy tended to have a lower 14-day mortality in patients with VAP, but this did not reach a statistical significance (OR 0.25, 95% CI 0.04–1.11, *p* = 0.09). In other subgroups stratified with age and the responsible pathogen, results were similar to the overall analyses. As polymicrobial infection and inappropriate initial antibiotic therapy might impact patients’ outcomes, subgroup analyses by excluding these patients did not change the trend of these results. Moreover, when only including patients with septic shock, the combination therapy was still not associated with decreased 14-day mortality (Table 3).

## 4. Discussion 

As mentioned in the introduction, although the combination of tigecycline and polymyxins has been widely used, clinical evidence supporting the effectiveness of this combination is limited. In some case report studies, the success of using this combination in treating infections caused by carbapenem-resistant *A. baumannii* was observed [33,34]. In contrast, case–control or cohort studies did not support such benefit of this combination when compared with polymyxin alone [18,19,20]. In China, tigecycline-based therapy is the mainstream regimen for treating infections caused by carbapenem-resistant Gram-negative bacteria [7]; therefore, it is imperative to know whether adding polymyxin B to tigecycline could result in better clinical outcomes than tigecycline-based therapy. However, such comparative studies are extremely limited. A comprehensive literature review revealed only one recently published retrospective study, in which the effectiveness of tigecycline-polymyxin B combination in the treatment of hospital-acquired pneumonia that caused by carbapenem-resistant Enterobacteriaceae or carbapenem-resistant *A. baumannii* was assessed in comparison with tigecycline-based therapy or polymyxin B-based therapy [35]. In this study, the authors found that the combination of polymyxin B and tigecycline was not superior to appropriate polymyxin B-based therapy and tigecycline-based therapy (HR 0.50, 95% CI 0.31–0.81, *p* = 0.004, HR 0.77, 95% CI 0.53–1.12, *p* = 0.169, respectively). The lack of benefit might be partially attributed to the low concentration of tigecycline, as stated by the authors. Therefore, we reported results from our present study to answer whether the high-dose tigecycline regime would contribute to better clinical outcomes in this combination. To the best of our knowledge, this is the first study assessing the effectiveness of the adjunctive therapy of polymyxin B to high-dose tigecycline in treating nosocomial pneumonia due to carbapenem-resistant *A. baumannii* and *K. pneumoniae*. The results indicated that the combination therapy was not associated with better clinical outcomes when compared with the high-dose tigecycline therapy. These findings are in line with previous studies that the combination therapy with antibiotics demonstrating in vitro synergistic effects might not be superior to the monotherapy in treating infections due to carbapenem-resistant Gram-negative bacteria [36,37,38]; for example, colistin plus vancomycin was recently claimed to work synergistically in carbapenem-resistant *A. baumannii*, but clinical evidence did not identify any benefit of this combination in comparison with colistin alone [39].

Evidence supporting the combination of tigecycline and polymyxins in treating carbapenem-resistant infections was mainly derived from in vitro studies [10,11,40]. In these studies, tigecycline and polymyxin B (or colistin) were usually used at a high concentration; several times higher than MICs of the tested pathogens [41,42,43]. When polymyxins were used at their approved dosage in clinical practice, the concentration would be much lower than that was used in those in vitro studies [44]. Combining the clinically achievable concentration of polymyxin B with tigecycline did not demonstrate any synergistic activity against carbapenem-resistant *A. baumannii in vitro* [45]. Moreover, in animal studies, the in vivo activity of colistin in combination with tigecycline was also inconsistent. In one study, antagonism was observed in 33.3 to 44.4% of the strains when colistin was added to tigecycline [46]; by contrast, no antagonism was observed in another study; instead, the combination demonstrated a synergistic effect in 75% of strains [47]. Taken together, it is not surprising to observe inconsistent results between in vitro, (in vivo) and clinical studies. 

It is of note that the tigecycline used in the present study was at its high-dose regimen. As demonstrated in in vitro studies, using the high-dose regimen resulted in better pharmacokinetic and pharmacodynamic parameters of tigecycline in plasma and the lung [25,48]. Clinical studies have also demonstrated promising outcomes in patients who received a high-dose of tigecycline. A study including critically ill patients with multidrug-resistant bacterial infections indicated that the high-dose tigecycline regimen was the only independent predictor of clinical cure in VAP patients [26]. A recent meta-analysis including 10 studies of 593 patients showed similar results; in the subgroup analysis, the high-dose tigecycline regimen was associated with decreased mortality in patients with HAP and VAP (OR 0.39, 95% CI 0.22–0.70, *p* = 0.002) [27]. In the present study, the mortality was much lower (24.5%) than that reported in other studies (46.1% to 57.1%) [4,49], further supporting the promising effectiveness of the high-dose regimen. 

Furthermore, the specific pharmacokinetic/pharmacodynamic profile of polymyxins in the lung might also contribute to the non-benefit of the combination therapy in the present study. Pharmacodynamic studies have indicated that even when using the highest tolerable dose of polymyxins, the concentration of polymyxins in the lung is likely to be below optimal for infecting strains unless MICs of these pathogens are well below the breakpoint [44,50]. Therefore, a higher dose of polymyxin is required to achieve sufficient antibiotic attainment in such infections. Moreover, using a loading dose of polymyxin is also recommended, as a recent study has demonstrated that it could improve patient’s survival rate 1.7 times higher than those who did not receive the loading dose [51]. However, the opposite situation in clinical practice is that doctors are likely to prescribe polymyxins at a dosage lower than the recommended to avoid dose-dependent side effects rather than using a higher dose [52]. As also demonstrated in the present study, polymyxin B was used at a fixed dosage of 750,000 IU per 12 h, lower than the 12,500 to 15,500 IU/kg per 12 h recommended in clinical guidelines [40], which might further diminish the benefit of the combination therapy in treating pneumonia due to carbapenem-resistant pathogens. 

To overcome these pharmacokinetic disadvantages of polymyxins in the lung, the inhalation of polymyxins was therefore recommended [28,40]. Compared with intravenous colistin, inhaled colistin resulted in higher concentrations in lung tissues and epithelial lining fluid. In mechanically ventilated critically ill patients, the inhalation of 80 mg colistimethate sodium every 8 h achieved sufficient concentrations of colistin up to 4 h (median 6.7 and 3.9 mg/L at 1 and 4 h after the inhalation), which were several times higher than the MIC breakpoint for *A. baumannii* and *K. pneumoniae* [53]. When aerosolized a higher dose of colistin (2 million IU of colistimethate sodium), the epithelial lining fluid concentrations were even higher (9.53–1137 mg/L) [54]. Therefore, adjunctive therapy with aerosolized colistin might lead to better clinical outcomes. Indeed, clinical studies have demonstrated that compared with intravenous polymyxin alone, intravenous plus aerosolized polymyxin was associated with better clinical outcomes in patients with pneumonia due to multidrug-resistant pathogens [55,56]. However, whether the adjunctive therapy of aerosolized colistin (or polymyxin B) to tigecycline could result in better results is still unclear. A recent retrospective study indicated that the adjunctive therapy of nebulized colistin to conventional intravenous antibiotics (36.5% were tigecycline) resulted in lower 14-day mortality than those patients who did not receive the nebulized colistin [57]. Moreover, compared with patients receiving placebo aerosols, patients receiving nebulized colistin experienced favorable microbiological outcomes in a randomized controlled trial [58]. Therefore, it is rational to hypothesize that the adjunctive therapy of aerosolized polymyxin B (or colistin) to intravenous high-dose tigecycline might function better than the high-dose tigecycline alone in treating nosocomial pneumonia due to carbapenem-resistant bacteria. However, as no clinical evidence directly supports it, further studies are warranted.

Another reason for clinicians to apply combination therapies is trying to lower the rate of the emergence of antimicrobial-resistant pathogens [59]. In clinical practices, the evolution of tigecycline resistance in multidrug-resistant Gram-negative bacteria was reported during tigecycline monotherapy [60,61]. As the mechanism against tigecycline and polymyxins are different [62,63,64], it is possible to minimize the selection of resistant strains by using the combination therapy. Indeed, from the results of an in vitro study, the combination of tigecycline and colistin reduced the mutant prevention concentration of tigecycline, and no strains lost their susceptibility to tigecycline during the combination therapy; by contrast, the MICs of tigecycline increased 4- to 32-fold when using tigecycline monotherapy [65]. However, concentrations of antibiotics used in the previous study were higher than the clinically achievable concentration with the current dosage regimen. When using the clinically achievable concentration of tigecycline and colistin, this combination did not curb the occurrence of antibiotic resistance [66]. Moreover, the clinical evolution of resistance to tigecycline during the combination therapy with tigecycline and polymyxin B has also been reported [67]. In the present study, tigecycline resistance emerged in both groups. In patients receiving tigecycline-based therapy, strains with tigecycline resistance were isolated from three patients (with MIC of 4, 8, and 8 mg/L), while in the tigecycline-polymyxin B group, tigecycline resistance was identified from two patients (with MIC of 4 and 16 mg/L). However, because the duration of antibiotic therapy and subsequent sampling time points varied significantly in each patient, we cannot draw any conclusions regarding the efficacy of the combination therapy in curbing the emergence of tigecycline resistance. Nevertheless, the results from the present study highlight the possibility of isolating tigecycline-resistant strains even when using the combination therapy, which subsequently might lead to treatment failure. In terms of polymyxin resistance, although we did not document such information in the present study, previous research has demonstrated clearly that colistin resistance would also emerge during the combination therapy course [68], further emphasizing the importance of antimicrobial resistance monitoring during clinical practice. However, whether this combination would reduce the frequency of the evolution of antibiotic resistance is still unknown and warrants further studies.

There are several limitations in the present study. First, the nature of the retrospective, single-center study with the small sample size diminished the statistical power. In the comparative effectiveness analysis, although we applied the propensity score matching by incorporating covariates that were believed to be associated with clinical outcomes, it is unlikely to consider all possible confounders, especially with a limited sample size. Second, although patients receiving ceftazidime-avibactam were excluded from the final analysis, most patients still received concomitant antibiotics in the present study, such as carbapenems, sulbactam, fluoroquinolone, and aminoglycosides, further complicating the interpretation of the results because these concomitant antibiotics were demonstrated to have in vitro synergistic effects with tigecycline and polymyxin B. Third, although we only included patients receiving tigecycline or polymyxin B within three days after the release of the result of the antimicrobial susceptibility testing, polymyxin B was usually administrated as the adjunctive therapy to tigecycline 2 to 3 days later. This delay could also impair the true effectiveness of polymyxin B in treating carbapenem-resistant pneumonia. Moreover, the underdosed polymyxin B used in the present study could also be contributing to the non-beneficial effect of the combination therapy. Fourth, the genomic backgrounds of the mechanism mediating carbapenem resistance in these included strains were unknown. Different genomic backgrounds might be associated with different virulence; therefore, it could cause distinct clinical outcomes under the same antibiotic regimens, further complicating the interpretation of the final results. Fifth, apart from nephrotoxicity commonly caused by polymyxin B, in the present study, we did not take the adverse events of tigecycline into consideration, such as hepatic injury, coagulation disorders, etc., which are the most frequently reported adverse events during tigecycline therapy and might impact patient’s clinical prognosis. Finally, the degree of bacteremia in the two groups was unknown due to the low rate of blood culture. As tigecycline and polymyxin B have demonstrated suboptimal serum concentrations, the lack of such information may underestimate the efficacy of the regimen in treating pneumonia, especially in a cohort with a high proportion of patients (87.3%) having septic shock. Despite these limitations, the results presented here could still help reduce the unnecessary use of the tigecycline-polymyxin combination in patients with carbapenem-resistant pneumonia, as polymyxins plus tigecycline was the second most frequently used regime in treating pneumonia caused by carbapenem-resistant Enterobacteriaceae (41%) and carbapenem-resistant *A. baumannii* (46.3%) in France, Greece, Israel, Italy, Kosovo, Slovenia, Spain, and selected hospitals in the USA [17]; In China, it is also the third most frequently used regimen in treating pneumonia caused by carbapenem-resistant *K. pneumoniae* (29.3%), even though the availability of polymyxins is limited in a considerable proportion of Chinese hospitals [7].

## 5. Conclusions

In conclusion, compared with high-dose tigecycline therapy, the combination of polymyxin B and high-dose tigecycline did not improve clinical outcomes in patients with nosocomial pneumonia due to carbapenem-resistant *A. baumannii* and *K. pneumoniae*. As polymyxin B used in the present study was underdosed, whether the combination with an adequate dose of polymyxin could result in better clinical outcomes is still unknown and worth further clinical trials. At the current stage, adding polymyxin B with the package insert recommended dosage to the high-dose tigecycline regimen is not encouraged.

## Figures and Tables

**Table 1:** Characteristics of patients with nosocomial pneumonia caused by carbapenem-resistant *A. baumannii* or *K. pneumoniae*.

Variable	Original Cohort			Propensity Score-Matched Cohort			
	Tigecycline- Polymyxin B, *n* = 68, 42%	Tigecycline, *n* = 94, 58%	*p*	Tigecycline- Polymyxin B, *n* = 51, 50%	Tigecycline, *n* = 51, 50%	*p*	Standardized Differences
Age, years, median [IQR]	63 [52, 74.25]	63 [51, 75.75]	0.764	67 [54, 76.5]	56 [49, 75.5]	0.235	0.238
Male Gender, n (%)	48 (70.6)	64 (68.1)	0.867	36 (70.6)	35 (68.6)	1.000	0.043
Preexisting Medical Conditions, n (%)							
Hypertension	26 (38.2)	44 (46.8)	0.354	20 (39.2)	21 (41.2)	1.000	0.04
Diabetes Mellitus	16 (23.5)	24 (25.5)	0.915	14 (27.5)	11 (21.6)	0.645	0.137
Chronic Heart Disease	16 (23.5)	14 (14.9)	0.233	13 (25.5)	7 (13.7)	0.212	0.3
Chronic Kidney Disease	4 (5.9)	3 (3.2)	0.660	3 (5.9)	3 (5.9)	1.000	<0.001
Chronic Liver Disease	5 (7.4)	8 (8.5)	1.000	2 (3.9)	6 (11.8)	0.269	0.295
Malignancy	10 (14.7)	17 (18.1)	0.722	8 (15.7)	11 (21.6)	0.611	0.152
History of Surgery	29 (42.6)	38 (40.4)	0.903	20 (39.2)	20 (39.2)	1.000	<0.001
Charlson Comorbidity Index, median [IQR]	3 [1, 4]	3 [1, 5]	0.506	4 [2, 6]	4 [2, 6]	0.935	0.027
Type of Pneumonia, n (%)			0.730			0.222	0.285
Hospital-acquired Pneumonia	28 (41.2)	35 (37.2)		23 (45.1)	16 (31.4)		
Ventilator-associated Pneumonia	40 (58.8)	59 (62.8)		28 (54.9)	35 (68.6)		
Pathogen, n (%)			0.26			0.828	0.086
*A. baumannii*	45 (66.2)	71 (75.5)		35 (68.6)	37 (72.5)		
*K. pneumonia*	23 (33.8)	23 (24.5)		16 (31.4)	14 (27.5)		
Polymicrobial Pneumonia, n (%)	7 (10.3)	14 (14.9)	0.533	5 (9.8)	5 (9.8)	1.000	<0.001
Concomitant Antibiotics, n (%)			0.23			0.253	0.651
Aminoglycoside	0 (0.0)	2 (2.4)		0 (0.0)	2 (4.3)		
Fluoroquinolone	1 (1.8)	2 (2.4)		0 (0.0)	0 (0.0)		
Carbapenems	27 (48.2)	21 (25.6)		21 (53.8)	15 (32.6)		
Piperacillin-Tazobactam	0 (0.0)	4 (4.9)		0 (0.0)	2 (4.3)		
Cefoperazone-Sulbactam	24 (42.9)	50 (61.0)		16 (41.0)	25 (54.3)		
Carbapenem plus Sulbactam	4 (7.1)	2 (2.4)		2 (5.1)	2 (4.4)		
Carbapenem plus Moxifloxacin	0 (0.0)	1 (1.2)		0 (0.0)	0 (0.0)		
Inappropriate Initial Antibiotic Therapy	33 (48.5)	58 (61.7)	0.132	26 (51.0)	28 (54.9)	0.843	0.079
Duration of antibiotic therapy, days, median [IQR]	16 [10, 25]	14 [10, 21]	0.378	16 [10, 25]	15 [10, 25.5]	0.730	0.011
Septic Shock, n (%)	60 (88.2)	70 (74.5)	0.049	46 (90.2)	43 (84.3)	0.553	0.177
Duration of Vasopressors, days, median [IQR]	12 [7, 21.25]	8 [4, 15]	0.03	12.5 [8, 21.75]	9 [5, 15]	0.063	0.286
SOFA score, median [IQR]	10 [7, 12]	8 [5, 11]	0.018	10 [7.5, 12]	9 [7.5, 12.5]	0.944	0.043

**Table 2:** Clinical outcomes in patients with nosocomial pneumonia caused by carbapenem-resistant *A. baumannii* or *K. pneumoniae*.

Clinical Outcomes	Patients Included in Analysis, No./Total No. (%)		
	Tigecycline- Polymyxin B	Tigecycline	Odds Ratio ^a^ (95% CI), *p* Tigecycline as Reference
**Overall analysis**			
14-day mortality	14/68 (20.6%)	23/94 (24.5%)	0.73 (0.32–1.62), 0.449
Clinical cure	39/68 (57.4%)	57/94 (60.6%)	0.95 (0.47–1.92), 0.895
Microbiological cure	19/68 (28.4%)	32/94 (34.0%)	0.84 (0.39–1.72), 0.627
Nephrotoxicity rate	31/68 (45.6%)	37/94 (39.4%)	0.91 (0.44–1.85), 0.792
**Matched cohort**			
14-day mortality	11/51 (21.6%)	14/51 (27.5%)	0.72 (0.27–1.83), 0.486
Clinical cure	28/51 (54.9%)	27/51 (52.9%)	1.09 (0.48–2.54), 0.823
Microbiological cure	13/51 (25.5%)	15/51 (29.4%)	0.96 (0.39–2.35), 0.928
Nephrotoxicity rate	24/51 (47.1%)	26/51 (51.0%)	0.85 (0.36–1.99), 0.712

^a^ The odds ratios were calculated by adjusting for the SOFA score, polymicrobial infection, inappropriate initial antibiotic therapy and the age-adjusted Charlson comorbidity index score. Area under the receiver operating characteristic curve for the multivariable logistic model of the primary outcome was 0.73 (95% CI, 0.63–0.83) in the original cohort and 0.71 (95% CI, 0.58–0.83) in the propensity score matched cohort, respectively. SOFA, Sequential Organ Failure Assessment; CI, confidence interval.

**Table 3:** Subgroup analysis of the primary outcome in patients with nosocomial pneumonia caused by carbapenem-resistant *A. baumannii* or *K. pneumoniae*.

Subgroup Analysis ^a^	OR (95% CI)	*p*
**Age**		
≤65	1.22 (0.21–7.21)	0.812
>65	0.40 (0.12–1.31)	0.137
**Pneumonia type**		
HAP	1.15 (0.31–4.47)	0.832
VAP	0.25 (0.04–1.11)	0.09
**Pathogen**		
*A. baumannii*	0.43 (0.13–1.28)	0.139
*K. pneumoniae*	2.72 (0.48–21.9)	0.283
**Initial empirical antibiotic therapy**		
Appropriate	0.39 (0.07–1.69)	0.221
Inappropriate	1.11 (0.34–3.62)	0.859
Excluded polymicrobial pneumonia	0.56 (0.21–1.44)	0.231
Excluded patients without septic shock	0.65 (0.25–1.65)	0.366

^a^ The subgroup analysis was conducted in the propensity score weighted cohort, unadjusted OR of the 14-day mortality in each subgroup was calculated by defining patients receiving tigecycline as the reference group. OR, odds ratio, CI, confidence interval, HAP, Hospital-acquired Pneumonia, VAP, Ventilator-associated Pneumonia.

## Data Availability

The datasets generated and analyzed during the current study are available from the first authors (Lei Zha and Xue Zhang) upon reasonable request.
